# Protocol for a multi-site study of the effects of overdose prevention education with naloxone distribution program in Skåne County, Sweden

**DOI:** 10.1186/s12888-020-2470-3

**Published:** 2020-02-07

**Authors:** Katja Troberg, Pernilla Isendahl, Marianne Alanko Blomé, Disa Dahlman, Anders Håkansson

**Affiliations:** 1 Department of Clinical Sciences, Psychiatry, Faculty of Medicine, Lund University, Lund, Sweden; 2 Division of Psychiatry, Addiction Center Malmö, Region Skåne, Malmö, Sweden; 3 Malmö Addiction Centre, Clinical Research Unit, Södra Förstadsg. 35, Plan 4, S-205 02 Malmö, Sweden; 4 Department of Infectious Disease, University Hospital Skåne, Malmö, Sweden; 5 Regional Office for Communicable Disease Control, Malmö, Sweden; 6 Department of Clinical Sciences, Center for Primary Health Care Research, Lund University/Region Skåne, Malmö, Sweden

**Keywords:** Take-home naloxone, Opioids, Needle exchange program, Opioid substitution treatment, Substance dependency

## Abstract

**Background:**

Continuously high rates of overdose deaths in Sweden led to the decision by the Skåne County to initiate the first regional take-home naloxone program in Sweden. The project aims to study the effect of overdose prevention education and naloxone distribution on overdose mortality in Skåne County. Secondary outcome measures include non-fatal overdoses and overdose-related harm in the general population, as well as cohort-specific effects in study participants regarding overdoses, mortality and retention in naloxone program.

**Methods:**

Implementation of a multi-site train-the-trainer cascade model was launched in June 2018. Twenty four facilities, including opioid substitution treatment units, needle exchange programs and in-patient addiction units were included for the first line of start-up, aspiring to reach a majority of individuals at-risk within the first 6 months. Serving as self-sufficient naloxone hubs, these units provide training, naloxone distribution and study recruitment. During 3 years, questionnaires are obtained from initial training, follow up, every sixth month, and upon refill. Estimated sample size is 2000 subjects. Naloxone distribution rates are reported, by each unit, every 6 months. Medical diagnoses, toxicological raw data and data on mortality and cause of death will be collected from national and regional registers, both for included naloxone recipients and for the general population. Data on vital status and treatment needs will be collected from registers of emergency and prehospital care.

**Discussion:**

Despite a growing body of literature on naloxone distribution, studies on population effect on mortality are scarce. Most previous studies and reports have been uncontrolled, thus not being able to link naloxone distribution to survival, in relation to a comparison period. As Swedish registers present the opportunity to monitor individuals and entire populations over time, conditions for conducting systematic follow-ups in the Swedish population are good, serving the opportunity to study the impact of large scale overdose prevention education and naloxone distribution and thus fill the knowledge gap.

**Trial registration:**

Naloxone Treatment in Skåne County - Effect on Drug-related Mortality and Overdose-related Complications, NCT 03570099, registered on 26 June 2018.

## Background

In individuals with opioid use disorder or misuse of opioids, opioid-related overdose is the worldwide leading cause of premature deaths. Compared to the general population, people using opioids in Europe are between five and ten times more likely to die than their peers of the same age and gender [1]. Northern Europe and Scandinavia are greatly affected by high rates of opioid-related overdose deaths, and Sweden has experienced a steady increase during the last decade accounting for the second highest rates in Europe [2, 3]. Opioid overdoses are, to a large extent, preventable as a significant part (50–96%) of heroin users reported witnessing at least one clinically well-defined overdose [4], leaving a window of opportunity to intervene. Brief overdose training has been shown to be sufficient [5] and bystanders are willing and able to engage in life saving action [6, 7]. Having no addictive or psychoactive effect, the opioid antagonist naloxone has no abuse potential, is safe and effective, if given soon enough after the occurrence of an opioid overdose. Naloxone reverses the negative effect of opioids on respiratory depression and decreased consciousness. Serious adverse effects are rare [8, 9]. In 2014, the World health organization recommended that naloxone should be made available for all individuals likely to witness an opioid overdose [10]. Over the past two decades, there has been an expansion of overdose prevention education and naloxone distribution (OPEND) programs training bystanders how to intervene during a witnessed opioid overdose. Studies have shown that take-home naloxone (THN), accompanied with education and training related to overdose management, may be associated with decreased overdose related mortality [9, 11] and has been described to be cost-efficient [12, 13]. The evidence is insufficient as the majority of previous studies have been observational, uncontrolled studies, based on the self-report of administered naloxone doses and the outcome of overdose victims. Also, a recent randomized controlled trial in the US setting did not demonstrate an effect on overdose events from naloxone distribution, in comparison to an education-only study arm. This study, however, was limited in size and used any overdose event (non-fatal) as outcome, but demonstrates the complexity of overdose interventions and the large need for more controlled intervention studies in the area [14]. Thus, there is need for large scaled controlled studies, measuring the impact of THN programs on opioid overdose death in the population, as these studies have been scarce. Reports from Norway showed that implementation of a large scale governmental THN program was feasible, successfully distributing a high-volume of naloxone by the use of an already existing network of facilities [15, 16]. Research in United States by Walley et al. demonstrated a significant decrease in opioid related deaths in communities providing OPEND, where the greatest impact was observed in communities distributing > 100 THN kits per 100,000 inhabitants [17].

In June 2018, Skåne County was the first region of Sweden to launch a multi-site OPEND program in Sweden, aiming to study the impact of naloxone prevention education and distribution on overdose mortality and overdose-related morbidity within the population. This protocol describes the implementation for monitoring the effects such a program.

### Risk factors for overdoses

Recent non-fatal overdose has been shown to represent a marker for increased risk of subsequent fatal overdose [18–20]. Recent period of abstinence, such as post-release from prison [21–24] or inpatient treatment [25–27], polydrug use [28–32], are risk factors highly associated with opioid overdose. Retention in opioid substitution treatment (OST) is associated with reduction of mortality and morbidity [33–39]. However, the OST population in Europe is ageing, with a mean age of death (39.4 years) increasing steadily [1], and carries a high degree of burden when it comes to chronic disease and multi-morbidity [29, 40] which also adds to the risks of overdose. Contrary to many beliefs, tolerant older users have been shown to have a higher risk of death due to overdose than novice users [28, 41].

### Opioid overdose

Defined as loss in consciousness and respiratory depression, opioid overdose is not a rare event among people who use opioids. Reports from Norway and Sweden have shown that the majority (> 70%) of heroin users have experienced at least one previous non-fatal overdose [16, 42, 43]. Estimations show that approximately one in 20 to one in 30 opioid overdoses leads to death [36]. Research describes an array of direct and indirect negative consequences of non-fatal opioid overdoses, such as pulmonary [44, 45] and cardiovascular complications [46], peripheral neuropathy [47], cognitive impairment [48] and rhabdomyolysis, which can lead to muscular problems, kidney injuries or failure [49]. Indirect injuries, such as injuries caused by falling, burns and assault, during loss of consciousness, are also rather frequently displayed [47].

As non-fatal overdoses are far more common than fatal opioid overdoses it is difficult to estimate the impact even of larger naloxone distribution programs. As non-fatal overdose commonly leads to both internal and external injuries it is important to focus both on reduction in mortality and reduction in morbidity. It is, however, impossible to predict whether a reversed opioid overdose would have been fatal, had naloxone not been administered, nor if potential injuries would have been avoided. This, the addition of societal factors that fluctuate over time, such as availability of drugs and potency of opioids, makes the estimated impact of OPEND on mortality and morbidity problematic. As research has shown that survival time exceeded 20–30 min in a majority of opioid related overdose fatalities, there is an opportunity for intervention [50]. By using naloxone, opioid overdoses can efficiently and safely be reversed by witnessing bystanders [51, 52].

### Routes of naloxone administration

Opioid receptor antagonist naloxone has been used in emergency and hospital settings since the United States Food & Drug Administration (FDA) approval in 1971, for intravenous, intramuscular and subcutaneous administration [53]. Subsequently, intranasal administration was suggested as a safe and efficient alternative to the use of needles. Injectable naloxone has been used off-label for intranasal administration throughout several years by prehospital staff [54–60], emergency medical services [61] and in the emergency department [62] with satisfactory clinical effect, compared to IV or IM administration [59, 60, 63]. Approved by the FDA in November 2015 [63], pharmacokinetic studies comparing the new intranasal to intra muscular administration, have shown satisfying results [64–67].

### Education and training

Bystanders are willing and able to respond appropriately to an overdose situation [6, 7, 68]. Naloxone training significantly improves knowledge regarding prevention and correctly managing opioid related overdoses [69–73]. Researchers also have suggested other benefits of naloxone and CPR training; Seal and co-workers, in a small study following patients during 6 months after CPR training and naloxone distribution, demonstrated that past-30-day heroin injecting decreased in the study participants [74]. Also, from qualitative interviews with clients trained in overdose intervention and naloxone administration, Wagner and co-workers described feelings of increased empowerment in the participants [75].

### Opioid overdose mortality in Sweden

Reported incidence of drug related death is approximately four times higher in Sweden (92 deaths/million) [3] compared to the average of Europe (22.6 deaths/million) [1]. Acute onset lethal intoxications have gradually increased since 1991, accompanied by an increase of deaths involving other opioids such as buprenorphine, fentanyl, methadone and tramadol [1, 76]. Toxicology records show that opioids are present in 95% of the drug related deaths. These records also show a high, and increasing, proportion of cases exhibiting the presence of more than one substance, indicating that poly-drug use, to a large extent, contributed to their death. The slight decrease in numbers of drug related deaths between 2015 and 2016 unfortunately proved to be a short break in the otherwise increasing numbers [1–3], showing that these numbers do fluctuate over time, adding to the challenging task of drawing conclusions from the result of interventions.

### Naloxone availability in Sweden

The National Board of Health and Welfare’s review of the legal situation established, in June 2017, that naloxone could be prescribed to people at risk of overdose, in relation to current legislation [77]. Prior to this, naloxone prescription was not legal in Sweden [78]. In the region studied here, the present study started soon after naloxone prescription was made legal. Prior to the study, distribution of naloxone in the region is likely to have been virtually non-existent. Naloxone distribution was already carried out in the capital area of Denmark [79], in close connection to the southernmost regions of Sweden, and few months before the start of the present project, distribution was initiated in the Stockholm region. Also, earlier than that, unauthorized distribution from peer support groups has been reported from the Stockholm area. Although a limited amount of naloxone potentially may have reached the region across the nation border or from a geographical distance from the Stockholm area, availability of naloxone in the present target region is likely to have been very low prior to study start.

Before approval of the new high concentration nasal spray containing 1.8 mg of naloxone hydrochloride for the use by laypersons, the prefilled vial with a solution of 0.4 mg/ml was approved and available to order from April 2018. The nasal spray was available in June 2018 for the Swedish market.

Naloxone is to be prescribed directly to the patient, given that the patient has received information about how to recognize an opioid overdose, how to administer naloxone and how to give life support while waiting for ambulance to arrive [77]. While relatives or friends of the patient can receive information and education as above, naloxone can only be prescribed to the patient at risk [77]. As a way to make THN more available, changes in the regulations, made in November 2018, allowed registered nurses, not only physicians, to prescribe naloxone [77]. In the beginning of 2019, The National Board of Health and Welfare strongly recommended regional health care facilities to offer THN to all individuals at risk of opioid overdose. Although prescription of naloxone is restricted, the accompanying information, on how to act and administer naloxone in case of an overdose, is general and addresses both individuals with substance use and individuals at risk of becoming a bystander in an overdose situation [80]. There are no legal constraints for non-users of opioids, such as family members or other potential bystanders, to use naloxone to reverse overdoses.

## Methods/design

### Setting

The present setting is the county of Skåne, the southernmost county of Sweden with around 1.3 million inhabitants. The major city of the region is the city of Malmö, with around 315,000 inhabitants, and three other major cities (Helsingborg, Lund, and Kristianstad) with populations of 145,000 to 85,000 inhabitants. By Swedish measures, the region of Skåne has unique access to both needle exchange programs (NEP) and opioid substitution treatment (OST) clinics through a network of units which enable reaching a large proportion of illicit drug users at risk of an opioid overdose. The Skåne region has seen a considerable expansion of OST in recent years, and in November 2017, a total of 1514 patients were enrolled in OST in the region. The region has four NEPs, in the four largest cities of the region, and which are open on weekdays and administered by the departments of infectious diseases within the regular hospital system. Altogether, the NEPs of the region have around 1000 to 1100 patients enrolled annually. The number of people at risk of an opioid overdose in the region is likely difficult to establish, as overdoses may occur both to patients known in addiction treatment or harm reduction, based on their opioid dependence, but also to unregular opioid users including patients seen in treatment for other addictive disorders and who may occasionally misuse opioids.

Overdoses in the present region have been examined in several papers during the past decade. For example, it was documented in qualitative interviews that despite an overall willingness from heroin users to help peers who overdose, several potential barriers against adequate interventions by bystanders were identified, such as uncertainty about the victim’s own intentions, the influence of one’s own intoxication, and fear of police when calling the ambulance [81]. Also, a smaller survey carried out in OST patients in the same region demonstrated high prevalence of overdoses, but also a very high interest in participating in a potential naloxone program [78].

### The Skåne naloxone project

In accordance with the decision of the Health Board in Skåne County in December 2014, the Director of Health was commissioned to monitor the possibilities of conducting a pilot study on THN with intranasal naloxone, and to report the case in March 2015. Skåne County has since followed the development of ongoing clinical studies and ongoing projects. In March 2015, The Director of Health was commissioned to start an experimental project as soon as possible in the form of a clinical study using intranasal naloxone spray. The pilot project was started after naloxone distribution was made legal by the authorities and after the nasal spray became available, and it is to be run for 3 years and during this time frame the scope of the study could be expanded if additional evidence was to be added [82].

Since prescription or distribution to a third party is prohibited by law, a well-structured and efficient multi-site implementation and distribution should provide the means to reach a large proportion of high-risk individuals with a relatively high likelihood of becoming bystanders at overdose situations. Implementation of this type of intervention setting where bystanders will administer naloxone and use life-supportive interventions as taught by the naloxone providers is believed to be feasible in a Swedish setting. The project was launched in the second quarter of 2018 [83]. The project is conducted by a steering group with representation from the organization of the county (Region Skåne) as well as the director of the clinical trials support organization of the region, as well as the principal investigator (the last author of the present paper), and the project leaders (the remaining authors of the paper). The current naloxone distribution program will continue for 3 years, and study outcomes will be followed for up to 5 years.

#### Study aims

The present study is a prospective cohort study testing the effect of an intervention, and comparing the effect in comparison to a historic comparison period. The primary aim is to study whether the project has an effect on overdose mortality in the general population in the region. Secondary outcome measures include the effect on incidence of non-fatal overdoses and ambulance-witnessed outcome measures of non-fatal overdoses in the general population, as well as outcomes specific to the included cohort participants receiving naloxone prescriptions, including their program retention, mortality and non-fatal overdose morbidity.

#### Methods

All NEPs (4, in the cities of Malmö, Lund, Helsingborg and Kristianstad) and OST clinics (18, in the cities of Malmö, Lund, Helsingborg, Kristianstad, Ystad, Ängelholm, Landskrona and Trelleborg) in the region were included in the project and in the research study, conducted by Lund University. Attempting to reach the population of individuals who have chosen not to visit NEP or enrol in OST, the project also included the emergency ward and the opioid detox ward at Malmö Addiction Centre, serving a majority of the population in Skåne. The goal is to offer OPEND to all individuals visiting targeted units, and to include 2000 individuals in the study. In the present setting, as from hospital records, NEP participants can be identified from their unique personal identification numbers, as NEP participation and interventions by NEP staff are documented. This enables systematic and longitudinal research including NEP patients in the present setting, including follow-up of NEP attendance over time. Several previous research studies from the present setting have originating from the identification of patients attending NEP units in the region [42, 84–86].

#### Primary outcome measures

Mortality due to opioid intoxication in the general population in Skåne County in 2019–2023, compared with historical mortality due to opioid intoxication during 2013–2017. Number of deaths will be collected from national registries.

#### Secondary outcome measures

##### Secondary outcome measures in the general population

- Reaction level of ambulance-attended opioid overdose survivors in the general population. Assessment of responsiveness in acute brain disorders using Reaction Level Scale (RLS-85) in ambulance. The RLS scale is graded from 1 (awake, no delayed reaction, oriented) to grade 8 (unconscious, no movements to painful stimuli) [Time Frame: 2019–2023].
- Respiratory rate of ambulance-attended opioid overdose survivors in the general population. Respiratory rate (breaths per minute) registered in ambulance [Time Frame: 2019–2023].
- Heart rate of ambulance-attended opioid overdose survivors in the general population. Heart rate (beats per minute) of opioid overdose survivors registered in ambulance [Time Frame: 2019–2023].
- Naloxone or other antidote administered by ambulance staff to ambulance-attended opioid overdose survivors in the general population [Time Frame: 2019–2023].
- Need of ambulance transport to hospital of ambulance-attended opioid overdose survivors in the general population, information registered in ambulance [Time Frame: 2019–2023].
- Incidence of opioid overdoses attended by ambulance or emergency hospital care (i.e. including non-fatal overdose cases), information registered in ambulance or from diagnostic codes registered in emergency hospital departments [Time Frame: 2019–2023].

##### Secondary outcome measures in included patients

- All-cause mortality in included patients. Number of deaths will be collected from national and regional registries [Time Frame: 3 years].
- Overdose mortality in included patients [Time Frame: 3 years].
- Retention in naloxone program - number of patients [Time Frame: 3 years].
- Incidence of witnessing opioid overdoses [Time Frame: 3 years].
- Incidence in naloxone use and bystander pulmonary resuscitation [Time Frame: 3 years].

#### Power and sample size

Power has been calculated only for the primary outcome measure. The total population at risk of fatal overdose in the general population is unknown, as people may be at risk of overdose death either because of a severe opioid dependence, a less severe state of dependence or a more unregular use of opioids. As the number of potentially affected individuals is unknown, the power calculations were based on numbers calculated from the number of individuals reached by the intervention at NEP and OST facilities. Thus, power calculations based on the cohort planned to receive distributed naloxone will be considered to be a minimum level with the possibility of a higher effect if the naloxone intervention disseminates also beyond the NEP and OST populations.

The number of patients in OST and NEP is estimated to comprise approximately 2000 individuals, 1000 individuals respectively. Power was calculated based on potential mortality in these two groups (calculated conservatively for 4 years prior to the intervention); a total of 4 × 2000 = 8000 person years; referred to as a ‘comparison period’), and with the conservative assumption that the intervention is calculated to reach the full study group for 1.5 years (intervention group, 2019–2020) (1.5 × 2000 = 3000 person years). The statistical power calculations are made with the Microsoft Excel sheet EPISHEET available for download at www.krothman.org/episheet.xls (downloaded on 14 January 2016).

Assuming that mortality before intervention is 50 deaths per 1000 person years in the overall cohort (that is, a 5% annual risk), a relative risk reduction of 25% (relative risk RR = 0.75) is required in order for the statistical strength to exceed 80% (81%), 5% significance limit, two-sided test, with 8000 person years before intervention (comparison period) and 3000 person years after intervention (intervention group). If however, assuming that mortality before intervention is 30 per 1000 person years in the overall cohort (that is, a 3% annual risk), a higher relative risk reduction, 32% (RR = 0.68), is required for the statistical strength to exceed 80% (81%), 5% significance limit, two-sided test, with 8000 person years before intervention (comparison period) and 3000 person years after intervention (intervention group). If the risk reduction still is 25%, even in this scenario, the statistical strength drops to 57%.

Based on previous studies from other types of settings, one can assume that, during follow-up, 10–20% of those who received naloxone have used these doses [87–89]. It is not possible to establish how many of those given naloxone, in an overdose situation, would have died if the dose had not been administered. It is also not possible to draw conclusions from the literature on how often a person with access to naloxone can be expected to be present when an overdose occurs. With a reduction in number of deaths (per 1000 person years) from 30 to 20 individuals, in regards to the power calculation above, and assuming that only 10% of those receiving naloxone use the dose, 100 (10 × 10) present overdose witnesses are required with access to naloxone for dose to be given in ten cases. With a careful estimate of how often an overdose will be witnessed by someone carrying, or having access to, naloxone, it is our belief that 2000 study participants, with a plan for inclusion of around 1000 patients from OST and 1000 patients from NEP, will be sufficient in order to detect significant reductions in mortality. In addition, the present calculations cautiously assume that the spread of naloxone will take place primarily within the NEP and OST populations. Although the individuals receiving naloxone in the program are either NEP or OST clients, it also should be borne in mind that theoretically, broader groups of individuals at risk of opioid overdoses may benefit from the intervention in case of naloxone spreading outside the core populations seen in the project.

In addition to the effects studied above, the correlation will be calculated between the number of distributed doses in the study cohort and the effects on the outcome variables above.

### Statistical methods

Comparison of mortality, before and after intervention, is done by Poisson regression with an indicator variable for the intervention (0 = comparison, 1 = intervention), adjusting for age and gender distribution. The effect is described as RR (relative risk), with 95% confidence intervals. *P* values < 0.05 will be considered as statistically significant. The primary outcome measure relates to mortality in the overall cohort. The effect on mortality will be studied in OST and NEP separately, using a subgroup analysis. The evidence in effect between the cohorts will be described by the *p*-value of an interaction test. Outcomes are studied year by year during the intervention period, compared to the comparison period, year by year.

For the study aims regarding the included cohort, this is open, dynamic cohort based on individuals in OST and NEP, where they contribute with person time, as at-risk from entry to study end or death, or by possible exit from the cohort (the earliest of these three events is the end date of the personal time calculation). In the general population analyses, 2013 to 2017 constitute the comparison period, while corresponding data, during 2019–2023, constitutes the intervention period.

Each person included in the follow-up will contribute a record (row) in the research database for each year this person is included in one of the two cohorts. In the row, in addition to each individual’s consecutive number, variables such as date, sex, age, cohort (1 = OST, 2 = NEP), intervention (0 = comparison, 1 = intervention), person time, date in the event of death, and status (alive/dead) at the end of each year, will be registered. In addition, based on public registers of the number of patients in OST, in the analyses it will be possible to control for fluctuations in OST availability (number of patients in methadone and buprenorphine treatment, respectively) in the region, as OST availability is likely to affect mortality rates; for example, based on national register data from the present setting, it has been shown that the risk of overdose mortality varies depending on OST status [90].

Significance, as above, is 5%, when applying a double-sided test. The target for the statistical strength is set at 80% for primary outcome. Secondary outcome and subgroup analyses have not been calculated in strength.

### Structure of implementation – the cascade model

Based on the training curriculums developed by the Harm Reduction Coalition [91], and in accordance with WHO guidelines (2014) [92], the naloxone education was developed by Practicum Clinical Skills Centre Malmö, in collaboration with the naloxone project leaders and by the guidance of the Danish Naloxone project RedLiv [79]. Participants from the User’s union in Skåne, the head of each unit and a key trainer, appointed by each unit, were represented at the naloxone workshop, November 2017. The session provided participants with the opportunity to receive information about the naloxone project and exchange opinions on the material and model of implementation. Before project start, an open information session about the naloxone project, was held, targeting members of the public and collaborators, such as social services, police and low threshold shelters.

The information material and material used in training curriculums was approved by the User’s union, Skåne, and by co-workers on various levels of the organisation, before the key-trainer sessions were held in 27th of March and 19th of April 2018. The compulsory three-hour training curriculums were held in Malmö, the largest town in the region. An extra key-trainer session was held at the NEP in Kristianstad. Each unit also received a CPR-dummy enabling practicing steps in education requiring practical exercise. The CPR-dummy had a replaceable face and lungs to ensure hygienic demands and to facilitate practical education when done in groups. The education material was sent to all key trainers, providing them with easy access material, with the purpose of key trainers educating their peers before project launch.

After initial training, before launching the project on the 11th of June 2018, all participating units were sent a link to an on-line booking site where the project leaders could be booked for on-site assistance during the first trainer-patient session.

Information and educational material for key trainers, staff, patients and their family and friends, who might benefit from OPEND, were provided by project leaders before launch at each unit. The project also provided the units with as many naloxone kits as each unit requested. The naloxone-kit - a small red bag, contains vinyl gloves, breathing mask, wipes, leaflet with “easy-to-use” instructions, a certificate for trainer/patient to sign after completing training session and a card with informing the overdose victim that they had received treatment with naloxone. The naloxone itself had to be requisitioned through the same system where all other medication is requisitioned, at the expense of the naloxone project. Every kit contains two single naloxone sprays, each dose delivering 1.8 mg naloxone as hydrochloride dehydrate.

**Fig. 1:**
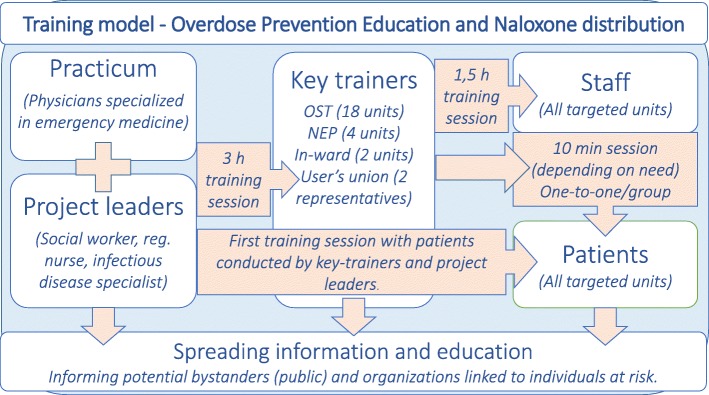
Training model OPEND Skåne Region – flowchart

OPEND training (Fig. 1) for users comprises a theoretical and a practical section. The theoretical section contains information on opioid receptors and effects of naloxone, risk factors and their possible impact on opioid overdose, how to identify and how to respond to an opioid overdose (is the person conscious/ breathing, call for ambulance, administer naloxone, airway management, recovery position, stay and observe consciousness and breathing). These steps are repeated as participants then practice on CPR dummy. Training lasts for 5–15 min, depending on trainee’s prior experiences and training. Sessions are held in groups or individually and can be extended if trainees request additional information or practical training to know more.

New staff are educated by key trainers, however project leaders are available for extra training sessions for staff and for key trainers. Open access information and education material is available on-line. Training can be provided by any member of staff who has completed the trainer education. Until November 2018, naloxone had to be prescribed by a physician, but can there-after be prescribed by a registered nurse. The naloxone kit is free of charge, and is provided to the trainee directly after training.

### Sample

#### Patient eligibility

First priority was to recruit patients from targeted units including 18 OST units, four NEPs and one emergency addiction unit and one in-patient detox ward for opioid dependence, serving under Addiction Center Malmö. Additional units will be eligible for inclusion when the project is implemented in prioritized units. Patients are eligible for the cohort study if they are 18 years and older, currently treated at the Addiction centres, or listed at NEP of Skåne County and have signed an informed consent. Exclusion criteria are restricted to subjects unable to understand study information due to psychiatric co-morbidity or severe language difficulties.

#### Recruitment

Recruitment began in June 2018 and will end in June 2021. On completion of the training session, patients are asked if they are interested in participating in the study. The trainer provides the patient with written and oral information about the study and how patient confidentiality will be protected. The trainer should also be able to answer questions raised by participants. If not, the project leaders are to be contacted without delay. Interested patients will then provide a written informed consent where upon they will be asked to fill in baseline questionnaire. Individuals declining study participation will access the same OPEND model as the individuals entering the study, and no compensation is given to the study subjects. The informed consent provided by included patients in the cohort study includes the follow-up in register data (national patient register and regional hospital and ambulance registers) for a three-year period, and register follow-up is carried out during the full three-year period, unless the patients contacts the researchers in order to withdraw consent. In case a patient ceases to show up in OST or NEP facilities in the region, only the self-report data regarding witnessed overdoses and naloxone administration will be dropped from further data collection.

#### Quantitative data collection

Included subjects in the cohort-specific analyses will be followed for 36 months. Questionnaires are obtained from initial training, on follow up, every sixth month, and upon THN refill, for any reason.

#### Measures

In order to distinguish acute drug-related deaths from deaths caused by diseases and organ damage caused by long-term use, and data from emergency and prehospital healthcare, collecting data on mortality, cause of death, and toxicological raw data, information will be gathered from the following registers:

- The National Board of Health’s Death Causes Register stores diagnostic information on mortality and causes of death such as international disease classification codes, ICD codes.
- The National drug index (linked to the National Board of Health’s death cause register), containing information on deceased persons, where drug addiction or drug poisoning is mentioned on the cause of death certificate, either as underlying or contributing cause.
- The National registry data for F1 diagnosis (ICD-10 - diagnoses related to harmful use or substance dependence) [89], for enabling interconnection of national mortality data with the presence of such diagnosis, in order to correctly identify deaths in individuals with substance use history
- The Public Health Agency’s research register Toxreg is based entirely on toxicological raw data, which is taken by routine when investigated by forensic medicine. Ninety three per cent of all unnatural / violent deaths among persons under the age of 65 are examined medically in Sweden, which means that the majority of all acute drug-related deaths can be found in Toxreg (the Public Health Authority, 2015).
- Skåne’s County medical and administrative registers for diagnoses and intervention codes indicating a condition of drug poisoning
- Data for all fatal and non-fatal overdose cases attended by ambulance in the region will be identified (including vital status of cases upon ambulance attendance) from a regional electronic documentation system for prehospital units in the Skåne County, where clinical data from ambulance-attended cases, and treatments given by ambulance staff, are documented. This data source covers the full intervention period in the study, but the electronic documentation system was introduced in early 2017, such that the comparison period can include this type of data only since then.
- Data from emergency hospital departments at hospitals in the region, describing all cases of overdose events who attend hospital (identified through diagnostic codes).

Regarding self-report data in the cohort study, surveys are administered to the research subjects during their visit to units, included in the project, distributing naloxone at six, 12, 18, 24, 30 and 36 months, respectively, and / or at re-supply (any cause). The enrolment data include information concerning demographics, own substance use during the last 30 days, previous experience of either witnessing an overdose and/or own experience of overdose and questions on recognition of, and handling of an overdose (*Questionnaire 1 see* Additional file 1).

Data collected upon naloxone refill concerns what happened to the previous naloxone/kit, for instance if it was stolen, lost, or used on oneself or on someone else. If it was used to reverse an overdose, questions on whom was it used and where did the overdose took place (private/or public space), will follow. Questions concern how to recognize an overdose, what to do if an overdose occurs to someone else, own substance use during the last 30 days, where naloxone is kept and if he/she sometimes are unaware of the content of their drugs, followed by statements concerning drug use, safety of naloxone, assessment of own risk behaviour and response to a potential or witnessed overdose (*Questionnaire 2–4 see* Additional files 2, 3 and 4).

### Data analysis

#### Data storage and missing data

Collected data from active intervention participants are registered in an electronic Case Report Form (e-CRF). A unique e-CRF is created for each active participant, separated from the identification of the study participant, and stored in the clinical trial software RedCap. Data are stored confidentially and cannot be accessed by anybody outside the study staff. All questions in the e-CRF are to be answered actively, if questions are not replied to, they shall be marked as NA = “Not applicable” or NK = “Not known”.

During the first 5 months data was collected on paper, as the use of the computerized application was delayed. In November 2018, each unit received their own iPad, which was provided for by the project, containing the RedCap application. Patient information and consent is still provided on paper. The 5 months of data collected on paper will be entered in the e-CRF by a research administrator at Lund University.

There will be no registration of individuals declining naloxone training. During the 3 years that the project will be running, individuals could have visited many of the sites, and have, most likely been asked on several occasions. However, aggregated data from OST and NEP facilities in the region will enable descriptions of whether included participants differ from these overall populations on a group level.

Statistical data are drawn from available data registers. Each individual included by follow up will contribute to the research database with one entry (row) for each year, in either cohort. During the follow up period, person-time time after cohort regression, will not be taken into account.

### Trial status

In July 2018, 3 weeks into the project, 70% of targeted units (*N* = 24) were included, providing OPEND and including subjects into the study. Five weeks into the project 87% of targeted units had started, after 12 weeks, all units were including patients in the project. After inclusion and establishment of the first targeted 24 units, making sure the program is well established into daily management, the naloxone project has recently included another 3 units, and are planning on further expansion. So far, reports from included units are indicating that upon completion of training, approximately 80% of patients are giving their consent to study inclusion. The intervention period begins 1 July 2019 (with the first analyses conducted for general population data for the 18-month period of 1 July 2019–31 December 2020), based on the assumption that the naloxone distribution will be considered to have reached a satisfactory level from July 2019.

## Discussion

This report describes the development, design and implementation of a regional, multi-site OPEND program in South Sweden, with the purpose to study the effects of a Naloxone distribution program in Skåne County. Building on existing infrastructure as a basis for the implementation was crucial, in order to manage rapid and safe implementation and expansion, targeting units reaching populations with highest risk for opioid overdoses, such as NEPs and OST clinics and units treating inpatients at high risk. Within 3 weeks 70% of targeted units were up and running and had started training patients. Within 12 weeks, all of the units were serving as OPEND units, including subjects to the study. Results are hypothesized to demonstrate the feasibility of implementing the first large scale OPEND in Sweden and to demonstrate an effect on population-based rates of opioid-related mortality from a large-scale naloxone distribution program.

### Opportunities and challenges

The unique infrastructure in the region makes Skåne County an interesting setting for implementation of a large scale naloxone program. From any town in the region, there is a possibility to access any of the four NEP’s or any of the 18 OST clinics within a maximum of one and a half hour by public transport. Although this setting is not found elsewhere in Sweden, this protocol, alongside all the education and information material produced in order to implement this project, shared on the project homepage [93], increases the ability for others to reproduce the study in another setting.

Distributing naloxone by and to peers and family members is not yet possible in Sweden, due to legislation, however training is. Increasing knowledge and awareness is a large part of making a change in the dark trends of high rates of overdose related deaths in Sweden. The goal set by the naloxone project, to educate all patients in OST and all patients visiting NEP, is high. However, if everyone who has the opportunity to obtain a kit does so and inform friends and family where they keep it, how to recognise and respond to an overdose, then there is a possibility to make a change. Naloxone projects offer the opportunity of actively working with attitudes and awareness connected to stigma as it depends on the collaboration between public health and the expert (the patient/user) [94], which has been found to increase empowerment among users [75].

The lack of formal THN programs to family members may be discussed as a potential limitation in future scientific publications from the project. Also, future documentation will assess and discuss the question of possible naloxone availability prior to study start, although this is suspected to be very low and likely to be close to negligible, as naloxone prescription became legal only soon before the project started.

In addition, further discussions in future publications can address if the settings included in the study, OST facilities, NEP facilities and emergency and detoxification wards in an addiction clinic, reach the full population of people with harmful use of opioids with risk of overdose. The NEP of the major city in the area, Malmö, has been described to reach around 70% of people who inject drugs in the catchment area of that facility [86], although such approximations are clearly difficult to make. However, OST and treatment centres also reach opioid-dependent subjects who do not inject. In addition, the future documentation of the study will address the limitation that individuals with prescription drug use on a level that does not cause any treatment or harm reduction contact may still be difficult to reach. Thus, all in all, it cannot be established how large the population at risk of overdoses in the region may be.

In relation to international targets, the absolute minimum annual naloxone distributed rate, should ideally be 9 to 20 times that of fatality rates, in order to prevent opioid overdose death [95]. Findings from research in Massachusetts suggest distribution of naloxone is dose related as the greatest reductions in opioid related deaths were seen in areas where distribution exceed 100 THN kits per 100,000 inhabitants [17]. Reaching all individuals at risk is certainly pivotal in order to efficiently fight drug related mortality and morbidity in the region, but this also relies on the same individuals to carry naloxone when there is a need for it. Self-reported carriage rate in the pilot study of N-ALIVE in the first 4 weeks after prison release was 71% [96]. Results from the national Scottish naloxone program show that even if there was a significant increase in prescriptions of naloxone, among participants accessing needle exchange services, disturbingly enough reported numbers on carriage showed a significant decrease over the same period, from 16 to 5 % [97]. A recent study in Norway, covering six cities with THN, showed 57% of subjects, recently reported heroin injection, currently carrying naloxone [98], numbers witnessing of a high degree of saturation. Differences in how carriage is defined should be taken into consideration, since this partially explain variations. Thus differences in actual carriage, and what motivates or discourages individuals to carry naloxone, needs to be further investigated.

Naloxone training provides the opportunity to increase knowledge and attitudes [73], shifting from sometimes dangerous and inappropriate folklore actions, to appropriately responding to opiate overdoses [99]. Increase of knowledge and attitude is also an important step in overdose prevention as false beliefs can have devastating consequences, such as high-risk individuals not perceiving themselves as being at risk [100]. The same remedy should be used for a constructive dialogue when responding to fears on over-antagonism [101] and fear of police involvement when calling the ambulance services [102–104]. Research shows that even after implementation of Good Samaritan Law, study participants still feared repercussions when calling ambulance services [102], while an overwhelming majority of participants from another study reported positive experiences of dealing with police and paramedics [105]. Real, or perceived as real, fears should be taken seriously as consequences of being controlled by fears can result in harm or, in worst case, death.

Though project funds cover all material and reimbursement for the naloxone spray, the project do not allocate additional resources or finances for compensation for time lost due to workforce training of colleagues and patients. Another challenge related to staffing was the decision to start implementation at the same time as the beginning of summer holidays. However, the priority was to get naloxone out on the streets as fast and as efficiently as possible. Waiting for a better opportunity would most likely equal a waste of valuable time to save lives.

## Supplementary information

**Additional file 1.** Naloxone training baselineR2.

**Additional file 2.** Refill own overdoseR2.

**Additional file 3.** Refill other than own overdoseR2.

**Additional file 4.** Followup or kit lost, stolen or given awayR2.

## Data Availability

The datasets used and/or analysed during the current study are available from the corresponding author on reasonable request, provided this is compliant with national legislation and with the decisions of the Swedish ethics committee.

## Abbreviations

CPR: Cardiopulmonary Resuscitation
e-CRF: electronic Case Report Form
FDA: Food & Drug Administration, United States
ICD: International Classification of Diseases
ICH GCP: International Conference on Harmonisation Good Clinical Practice
ITT: Intention To Treat
NA: Not applicable
NEP: Needle Exchange Program
NK: Not known
OPEND: Overdose Prevention Education and Naloxone Distribution
OST: Opioid Substitution Therapy
RLS: Reaction Level Scale
RR: Relative Risk
THN: Take-Home Naloxone
