# Supplementary material for: Protocol for a multi-site study of the effects of overdose prevention education with naloxone distribution program in Skåne County, Sweden
Source: BMC Psychiatry. 2020 Feb 7;20:49. doi: 10.1186/s12888-020-2470-3 (PMC7006080; doi:10.1186/s12888-020-2470-3)
Supplement: Supplementary file 1 — Additional file 1. Naloxone training baselineR2. [file 12888_2020_2470_MOESM1_ESM.docx]

| Name  **For initial naloxone training & kit: Form 1**  Naloxone refill, used to revers own OD: Form 2  Naloxone refill, used to reverse OD, other than my own: Form 3  Follow up, after X months / or kit lost/stolen/given to other: Form 4 |  | | | Date | |  | | |
| --- | --- | --- | --- | --- | --- | --- | --- | --- |
| Personal ID |  | | | Initial training at | | *(place)* | | |
| Mobile no |  | | | Follow-up at | | *(place)* | | |
| Follow-up coincides with Re-fill: | | Follow up at X months after initial training | | | | | | |
| No  O | Yes  O | 6  O | 12  O | 18  O | 24  O | | 30  O | 36  O |

X *Tick for the most appropriate alternative (-s)*

| **1. a) Have you, yourself, suffered an opioid overdose?** (i.e. opioid intake that has required either medical treatment or intervention by bystanders, due to unconsciousness or respiratory failure). | | | | | | | | | | | | | | | | |  |  |
| --- | --- | --- | --- | --- | --- | --- | --- | --- | --- | --- | --- | --- | --- | --- | --- | --- | --- | --- |
| O | Yes, at _________ number of occasions, last occasion being ______________ (date) | | | | | | | | | | | | | | | |  |  |
| O | No 🡪 # 2 O Don’t know 🡪 # 2 | | | | | | O | Don’t want to respond 🡪 # 2 | | | | | | | | |  |  |
|  | | | | | | | | | | | | | | | | |  |  |
| **b) What do you think was the reason behind your most recent overdose?**  *(Multiple answers possible)* | | | | | | | | | | | | | | | | |  |  |
| O | I just wanted to get well | | | | | |  | O Release from detox/incarceration/abstinence | | | | | | | | |  |  |
| O | My goal was to maximize the effect | | | | | |  | O Other, *please describe*: | | | | | | | | |  |  |
| O | I didn’t want to live | | | | | |  |  | | | | | | | | |  |  |
|  | | | | | | | | | | | | | | | | |  |  |
| 1. **Do you know what substances were used previous to the overdose?**  *(Multiple answers possible)* | | | | | | | | | | | | | | | | | |  |
| Heroin | | O | No | O | Yes | Cocaine | | | O | | No | | O | Yes | | | | |
| Benzodiazepines | | O | No | O | Yes | Pregabalin | | | O | | No | | O | Yes  Sleeping pills such as:  Zolpidem/  Zaleplon  Zopiclone/  Imovane | | | | |
| Alcohol | | O | No | O | Yes | Sleeping pills | | | O | | No | | O | Yes | | | | |
| Fentanyl | | O | No | O | Yes | Other:_________________________________ | | | | | | | | | | | |  |
| Buprenorphine | | O | No | O | Yes | Don’t know | | | O | |  | |  |  | | | | |
| Methadone | | O | No | O | Yes | No answer | | | O | |  | |  |  | | | | |
|  | | | | | | | | | | | | | | | | | |  |
| 1. **Have you ever witnessed someone else’s overdose?** | | | | | | | | | | | | | | | | |  |  |
| O | Yes, on _________ occations | | | | | | O | No | | O | | Don’t know | | | O | No answer | |  |
|  | | | | | | | | | | | | | | | | |  |  |
| 1. **How do you recognize an opioid overdose?** The person is/ has… *(Multiple answers possible)* | | | | | | | | | | | | | | | | |  |  |
| O | Snore-like gurgling noise/ choking sounds/ Irregular/shallow/slow, or no breathing | | | | | | O | Pale or clammy face, pulse (heartbeat) is slow, erratic, or not there at all | | | | | | | | |  |  |
| O | Fingernails and lips turn blue or purplish black | | | | | | O | Loss of consciousness, unresponsive to outside stimulus | | | | | | | | |  |  |
|  | | | | | | | | | | | | | | | | |  |  |
| 1. **How would you respond to an opioid overdose?** *I would…* *(Multiple answers possible)* | | | | | | | | | | | | | | | | |  |  |
| O | Call 112, for ambulance | | | | | | | | | | | | | | | |  |  |
| O | Administer naloxone | | | | | | | | | | | | | | | |  |  |
| O | Preform rescue breathing | | | | | | | | | | | | | | | |  |  |
| O | Place in stable position (when person is recovering) | | | | | | | | | | | | | | | |  |  |
| O | Stay with the person until ambulance arrives | | | | | | | | | | | | | | | |  |  |
| O | Other (please describe): | | | | | | | | | | | | | | | |  |  |

|  | |  | | | | | | | | | | | | | | | | | |  |
| --- | --- | --- | --- | --- | --- | --- | --- | --- | --- | --- | --- | --- | --- | --- | --- | --- | --- | --- | --- | --- |
| 1. **a) Own use last 30 days:** *(Multiple answers possible)* | | | | | | | | | | | | | | | | | | | |  |
| Heroin | | | | O | | No | O | Yes | | Cocaine | | | | | O | | No | O | Yes | |
| Benzodiazepines | | | | O | | No | O | Yes | | Pregabalin | | | | | O | | No | O | Yes | |
| Alcohol | | | | O | | No | O | Yes | | Sleeping pills | | | | | O | | No | O | Yes | |
| Fentanyl | | | | O | | No | O | Yes | | Other:_________________________________ | | | | | | | | | | |
| Buprenorphine | | | | O | | No | O | Yes | | Don’t know | | | | | O | |  |  | Sleeping pills such as:  Zolpidem/  Zaleplon  Zopiclone/  Imovane | |
| Methadone | | | | O | | No | O | Yes | | No answer | | | | | O | |  |  |  | |
|  | | | | | | | | | | | | | | | | | | | | |
| **b) Are you frequently mixing opioids with other substances for increased effect?** | | | | | | | | | | | | | | | | | | | |  |
| O | Not relevant, I don’t use opioids | | | | | | | | | |  |  | | | | | | | |  |
| O | Yes, with… 🡪 | | | | **c)** | | | | | |  |  |  | |  | | | | |  |
|  | | | | | Benzodiazepines | | | | | | O | No | O | | Yes | | | | |  |
|  | | | | | Sleeping pills | | | | | | O | No | O | | Yes | | | | |  |
|  | | | | | Alcohol | | | | | | O | No | O | | Yes | | | | |  |
|  | | | | | Cocaine/ Amphetamine | | | | | | O | No | O | | Yes | | | | |  |
|  | | | | | ADHD-pharma | | | | | | O | No | O | | Yes | | | | |  |
|  | | | | | Other | | | | | | O | No | O | | Yes 🡪 Which substance (-s)? _________ | | | | |  |
|  | | | | | Do not want to reply | | | | | | O | ___________________________________ | | | | | | | |  |
|  | | | | | | | | | | | | | | | | | | | |  |
| 1. **Are you sometimes uncertain of the contents of the drugs you’re using?** | | | | | | | | | | | | | | | | | | | |  |
| O | | Yes | O | | No | | | O | I don’t use drugs | | | | | O | | I don’t want to reply | | | |  |
|  | | | | | | | | | | | | | | | | | | | |  |
| 1. **Do you feel certain of what you should do in case of witnessing an overdose?** | | | | | | | | | | | | | | | | | | | |  |
| O | | Yes | O | | No | | | O | Other*, please describe*:­­­­­­ | | | | | | | | | | |  |
|  | | | | | | | | | | | | | | | | | | | |  |
| **Comments:** | | | | | | | | | | | | | | | | | | | |  |
