# Supplementary material for: Protocol for a multi-site study of the effects of overdose prevention education with naloxone distribution program in Skåne County, Sweden
Source: BMC Psychiatry. 2020 Feb 7;20:49. doi: 10.1186/s12888-020-2470-3 (PMC7006080; doi:10.1186/s12888-020-2470-3)
Supplement: Supplementary file 3 — Additional file 3. Refill other than own overdoseR2. [file 12888_2020_2470_MOESM3_ESM.docx]

| Name  For initial naloxone training & kit: Form 1  Naloxone refill, used to revers own OD: Form 2  **Naloxone refill, used to reverse OD, other than my own: Form 3**  Follow up, after X months / or kit lost/stolen/given to other: Form 4 |  | | | Date | |  | | |
| --- | --- | --- | --- | --- | --- | --- | --- | --- |
| Personal ID |  | | | Initial training at | | *(place)* | | |
| Mobile no |  | | | Follow-up at | | *(place)* | | |
| Follow-up coincides with Re-fill: | | Follow up at X months after initial training | | | | | | |
| No  O | Yes  O | 6  O | 12  O | 18  O | 24  O | | 30  O | 36  O |

X *Tick for the most appropriate alternative (-s)*

| 1. **Did you use your naloxone kit?** | | | | | | | | | | | | | | | | | | | | | | | | | | | | | | | | | | | | | |  | | | | | | | | | | | | |
| --- | --- | --- | --- | --- | --- | --- | --- | --- | --- | --- | --- | --- | --- | --- | --- | --- | --- | --- | --- | --- | --- | --- | --- | --- | --- | --- | --- | --- | --- | --- | --- | --- | --- | --- | --- | --- | --- | --- | --- | --- | --- | --- | --- | --- | --- | --- | --- | --- | --- | --- |
| O | | Yes, it was used to reverser an opioid overdose on 🡪 | | | | | | | | | | | | | | | | | | | | | | | O | | | | Me 🡪 Please fill out **Form** **2** (Refill own OD)! | | | | | | | | | | | | | | | | | | | | | |
|  | |  | | | | | | | | | | | | | | | | | | | | | | | O | | | | Man | | | | |  | | Whom is… | | | | | |  | | | | O | Relative | | | |
|  | |  | | | | | | | | | | | | | | | | | | | | | | | O | | | | Woman | | | | |  |  |  |  |  |  |  |  |  |  |  |  | O | Friend | | | |
|  | | | | | | | | | | | | | | | | | | | | | | | | | O | | | | Other | | | | |  |  |  |  |  |  |  |  |  |  |  |  | O | Acquaintance | | | |
| O | | No🡪Please fill out **Form 4** for follow-up! | | | | | | | | | | | | | | | | | | | | | | | | | | | | | | | | | | | | | | | | | |  | | O | Stranger | | | |
|  | |  | | | | | | | | | | | | | | | | | | | | | | | | | | | | | | | | | | | | | | | | | |  |  | O | Other: | | | |
| 1. **How did you know that this was an overdose?** The person had/ was… *(Multiple answers possible)* | | | | | | | | | | | | | | | | | | | | | | | | | | | | | | | | | | | | | | | | | | | | | | | | | | |
| O | | Snore-like gurgling noise/ choking sounds/ Irregular/shallow/slow, or no breathing | | | | | | | | | | | | | | | | | | | | | | | | | | | | O | | Pale or clammy face, pulse (heartbeat) is slow, erratic, or not there at all | | | | | | | | | | | | | | | | | | |
| O | | Fingernails and lips turn blue or purplish black | | | | | | | | | | | | | | | | | | | | | | | | | | | | O | | Loss of consciousness, unresponsive to outside stimulus | | | | | | | | | | | | | | | | | | |
|  | | | | | | | | | | | | | | | | | | | | | | | | | | | | | | | | | | | | | | | | | | | | | | | | | | |
| 1. **Where did the overdose occur?** | | | | | | | | | | | | | | | | | | | | | | | | | | | | | | | | | | | | | | | | | | | | | | | | | | |
| O | | In own accommodation | | | | | | | | | | | | | | | | | | | | | | | | | | | | O | | Other *(Please describe)*:­­­­­­ | | | | | | | | | | | | | | | | | | |
| O | | In someone elses’ accommodation | | | | | | | | | | | | | | | | | | | | | | | | | | | | O | | Don’t want to reply | | | | | | | | | | | | | | | | | | |
| O | | In a public place | | | | | | | | | | | | | | | | | | | | | | | | | | | |  | |  | | | | | | | | | | | | | | | | | | |
|  | | | | | | | | | | | | | | | | | | | | | | | | | | | | | | | | | | | | | | | | | | | | | | | | | | |
| 1. **Do you know what substances were used previous to the overdose?**  *(Multiple answers possible)* | | | | | | | | | | | | | | | | | | | | | | | | | | | | | | | | | | | | | | | | | | | | | | | | |  |  |
| Heroin | | | | | | | O | | | | No | | | O | | | | | Yes | | | Cocaine | | | | | | | | | | | | | O | | | | No | | | | O | | Yes  Sleeping pills such as:  Zolpidem/  Zaleplon  Zopiclone/  Imovane | | | |  |  |
| Benzodiazepines | | | | | | | O | | | | No | | | O | | | | | Yes | | | Pregabalin | | | | | | | | | | | | | O | | | | No | | | | O | | Yes | | | |  |  |
| Alcohol | | | | | | | O | | | | No | | | O | | | | | Yes | | | Sleeping pills | | | | | | | | | | | | | O | | | | No | | | | O | | Yes | | | |  |  |
| Fentanyl | | | | | | | O | | | | No | | | O | | | | | Yes | | | Other:_________________________________ | | | | | | | | | | | | | | | | | | | | | | | | | | |  |  |
| Buprenorphine | | | | | | | O | | | | No | | | O | | | | | Yes | | | Don’t know | | | | | | | | | | | | | O | | | |  | | | |  | |  | | | |  |  |
| Methadone | | | | | | | O | | | | No | | | O | | | | | Yes | | | No answer | | | | | | | | | | | | | O | | | |  | | | |  | |  | | | |  |  |
|  | | | | | | | | | | | | | | | | | | | | | | | | | | | | | | | | | | | | | | | | | | | | | | | | | | |
| 1. **a) How did you respond when witnessing the overdose?** *(Multiple answers possible)* | | | | | | | | | | | | | | | | | | | | | | | | | | | | | | | | | | | | | | | | | | | | | | | | | | |
| Called 112, for ambulance | | | | | | | | | | | | | | | | | | O | | | Yes | | | O | | No 🡪 | | | | | | | | **b)** *the reason being (for not calling 112):* | | | | | | | | | | | | | | | | |
|  | | | | | | | | | | | | | | | | | |  | | |  | | |  | |  | | | | | | | | O | | I was afraid that the police would turn up | | | | | | | | | | | | | | |
| Gav naloxon | | | | | | | | | | | | | | | | | | O | | | Yes | | | O | | No | | | | | | | | O | | I didn’t think it was necessary | | | | | | | | | | | | | | |
| Lungräddning (inblåsningar) | | | | | | | | | | | | | | | | | | O | | | Yes | | | O | | No | | | | | | | | O | | The one affected didn’t want me to call | | | | | | | | | | | | | | |
| Placerade i stabilt sidoläge | | | | | | | | | | | | | | | | | | O | | | Yes | | | O | | No | | | | | | | | O | | Fear that social welfare would be contacted | | | | | | | | | | | | | | |
| Stannade hos personen tills ambulans kom | | | | | | | | | | | | | | | | | | O | | | Yes | | | O | | No | | | | | | | | O | | Other, please describe: | | | | | | | | | | | | | | |
| Annat | | | | | | | | | | | | | | | | | | O | | | Yes,  please describe: | | | | | | | | | | | | | | | | | | | | | | | | | | | | | |
|  | | | | | | | | | | | | | | | | | | | | | | | | | | | | | | | | | | | | | | | | | | | | | | | | | | |
| 1. Witnessing someone suffering from an overdose can be a traumatic experience - **would you like to talk to someone about your experience**? **Önskar du prata med någon om din upplevelse?** | | | | | | | | | | | | | | | | | | | | | | | | | | | | | | | | | | | | | | | | | | | | | | | | | | |
| O | | Yes | | | O | | | | No | | | | | | | | | | | | O | | | Other, please describe: | | | | | | | | | | | | | | | | | | | | | | | | | | |
|  | | | | | | | | | | | | | | | | | | | | | | | | | | | | | | | | | | | | | | | | | | | | | | | | | | |
| 1. **a) Eget bruk de senaste 30 dagarna:** *(Multiple answers possible)* | | | | | | | | | | | | | | | | | | | | | | | | | | | | | | | | | | | | | | | | | | | | | | | | | | |
| Heroin | | | | | | O | | | | | No | | | O | | | Yes | | | | | Cocaine | | | | | | | | | | | | | O | | | | No | | | | O | | Yes | | | |  |  |
| Benzodiazepines | | | | | | O | | | | | No | | | O | | | Yes | | | | | Pregabalin | | | | | | | | | | | | | O | | | | No | | | | O | | Yes | | | |  |  |
| Alcohol | | | | | | O | | | | | No | | | O | | | Yes | | | | | Sleeping pills | | | | | | | | | | | | | O | | | | No | | | | O | | Yes | | | |  |  |
| Fentanyl | | | | | | O | | | | | No | | | O | | | Yes | | | | | Other:_________________________________ | | | | | | | | | | | | | | | | | | | | | | | | | | |  |  |
| Buprenorphine | | | | | | O | | | | | No | | | O | | | Yes | | | | | Don’t know | | | | | | | | | | | | | O | | | |  | | | |  | | Sleeping pills such as:  Zolpidem/  Zaleplon  Zopiclone/  Imovane | | | |  |  |
| Methadone | | | | | | O | | | | | No | | | O | | | Yes | | | | | No answer | | | | | | | | | | | | | O | | | |  | | | |  | |  | | | |  |  |
|  | | | | | | | | | | | | | | | | | | | | | | | | | | | | | | | | | | | | | | | | | | | | | | | | |  |  |
| **b) Are you frequently mixing opioids with other substances for increased effect?** | | | | | | | | | | | | | | | | | | | | | | | | | | | | | | | | | | | | | | | | | | | | | | | | | |  |
| O | | | Not relevant, I don’t use opioids | | | | | | | | | | | | | | | | | | | | | O | | | | Nej | | | | | | | | | | | | | | | | | | | |  |  |  |
| O | | | Yes, with… 🡪 | | | | | | | **c)** | | | | | | | | | | | | | |  | | |  | | | | |  | | |  | | | | | | | | | | | | |  |  |  |
|  | | | | | | | | | | Benzodiazepines | | | | | | | | | | | | | | O | | | No | | | | | O | | | Yes | | | | | | | | | | | | |  |  |  |
|  | | | | | | | | | | Sleeping pills | | | | | | | | | | | | | | O | | | No | | | | | O | | | Yes | | | | | | | | | | | | |  |  |  |
|  | | | | | | | | | | Alcohol | | | | | | | | | | | | | | O | | | No | | | | | O | | | Yes | | | | | | | | | | | | |  |  |  |
|  | | | | | | | | | | Cocaine/ Amphetamine | | | | | | | | | | | | | | O | | | No | | | | | O | | | Yes | | | | | | | | | | | | |  |  |  |
|  | | | | | | | | | | ADHD-pharma | | | | | | | | | | | | | | O | | | No | | | | | O | | | Yes | | | | | | | | | | | | |  |  |  |
|  | | | | | | | | | | Other | | | | | | | | | | | | | | O | | | No | | | | | O | | | Yes 🡪 Which substance (-s)? _________ | | | | | | | | | | | | |  |  |  |
|  | | | | | | | | | | Do not want to reply | | | | | | | | | | | | | | O | | | ___________________________________ | | | | | | | | | | | | | | | | | | | | |  |  |  |
|  | | | | | | | | | | | | | | | | | | | | | | | | | | | | | | | | | | | | | | | | | | | | | | | |  |  |  |
| 1. **Where do you keep your naloxon/kit?** | | | | | | | | | | | | | | | | | | | | | | | | | | | | | | | | | | | | | | | | | | | | | | | | |  |  |
| O | | | On me/with me | | | | | | | | | | O | | | At home | | | | | | | | | | | | | | | O | | Other (*please describe*): | | | | | | | | | | | | | | | |  |  |
|  | | | | | | | | | | | | | | | | | | | | | | | | | | | | | | | | | | | | | | | | | | | | | | | | |  |  |
| 1. **Are you sometimes uncertain of the contents of the drugs you’re using?** | | | | | | | | | | | | | | | | | | | | | | | | | | | | | | | | | | | | | | | | | | | | | | | | | |  |
| O | | | Yes | O | | | No | | | | | | | | | | | | O | | I don’t use drugs | | | | | | | | | | | | | | | | O | | | | I don’t want to reply | | | | | | | | |  |
|  | | | | | | | | | | | | | | | | | | | | | | | | | | | | | | | | | | | | | | | | | | | | | | | | | |  |

|  | | | | | |  |  |
| --- | --- | --- | --- | --- | --- | --- | --- |
| 1. Compared with **before** naloxone training – **How accurate are the following statements when it comes to your situation today?** |  | | | |  | |  |
|  | **Completly**  **accurate** | **Almost completly** | **Partly accurte** | **Not at all accurate** | **Not**  **relevant** | |  |
| I’m more careful when it comes to mixing drugs today | O | O | O | O | O | |  |
| I dare to take larger doses now when I have access to naloxone | O | O | O | O | O | |  |
| I’ve thought about switching to smoking heroine rather than injecting, since the latter is more safe | O | O | O | O | O | |  |
| I never try ”new drugs” when I’m by myself | O | O | O | O | O | |  |
| I’m more careful about quantity and quality of the drug when I haven’t used regularly (i.e. after detox/arrest) | O | O | O | O | O | |  |
| I know that I’m not as resilient when I’m sick (i.e. have an infection) as when I’m healthy | O | O | O | O | O | |  |
| I’m continuing to inject substances meant for oral use, (i.e. tablets or methadone for oral use) | O | O | O | O | O | |  |
| I know that my day-to-day health matters when it comes to how much my body can handle when it comes to drugs and other substances | O | O | O | O | O | |  |
| If administering naloxone, I’m afraid of spoiling someone’s “high” | O | O | O | O | O | |  |
| If witnessing an overdose, I will call an ambulance straight away | O | O | O | O | O | |  |
| It feels good being able to help someone who have overdosed | O | O | O | O | O | |  |
| I’m afraid of making mistakes when distributing naloxone | O | O | O | O | O | |  |
| I can easily identify if someone overdosed | O | O | O | O | O | |  |
| I’ve informed my friends and family where I keep my naloxone and how to use it. | O | O | O | O | O | |  |
| I feel certain that I know what to do in case of witnessing an overdose | O | O | O | O | O | |  |
| My view of myself has changed, I can save lives! | O | O | O | O | O | |  |
| I feel more of a responsibility now that I have the opportunity to save lives. | O | O | O | O | O | |  |
| **Comments:** | | | | | | | |
